# A novel PTRH2 missense mutation causing IMNEPD: a case report

**DOI:** 10.1038/s41439-021-00147-9

**Published:** 2021-06-10

**Authors:** Hossein Jafari Khamirani, Sina Zoghi, Mehdi Dianatpour, Aria Jankhah, Seyed Sajjad Tabei, Sanaz Mohammadi, Seyed Alireza Dastgheib

**Affiliations:** 1 Department of Medical Genetics, Shiraz University of Medical Sciences, Shiraz, Iran; 2 Student Research Committee, Shiraz University of Medical Sciences, Shiraz, Iran; 3 Stem Cells Technology Research Center, Shiraz University of Medical Sciences, Shiraz, Iran; 4 Shiraz Genetic Counseling Center, Shiraz, Iran; 5 Comprehensive Medical Genetic Center, Shiraz University of Medical Sciences, Shiraz, Iran

**Keywords:** Movement disorders, Neuromuscular disease

## Abstract

*PTRH2* deficiency is associated with an extremely rare disease, infantile-onset multisystem neurologic, endocrine, and pancreatic disease (IMNEPD). We report the first Iranian patient with IMNEPD. We detected a pathogenic variant in the *PTRH2* gene (NM_016077.5: c.68T > C, p.V23A). The proband has myopia, spastic diplegic cerebral palsy, urolithiasis, and a history of seizures.

Peptidyl-tRNA hydrolase 2 (PTRH2, Bcl-2 inhibitor of transcription 1) is a highly conserved protein encoded by *PTRH2* that prevents the accumulation of dissociated peptidyl-tRNA. It also promotes caspase-independent apoptosis. PTRH2 is located in the mitochondria, where it participates in protein synthesis via its hydrolase activity^1–3^. Provocation of cell death by induction of anoikis and activation of the PI3K-AKT-NFkB pathway and Bcl-2 transcription by PTRH2 have also been reported.

Disruption in the function of PTRH2 leads to infantile-onset multisystem neurologic, endocrine, and pancreatic disease, which was initially investigated and reported by Hu et al. and Alazami et al.^4,5^.

This paper reports a novel pathogenic variant of *PTRH2* (NM_016077.5: c.68T > C, p.V23A) that resulted in an interesting clinical presentation in a proband harboring two copies of the variant (Fig. 1).

**Fig. 1:**
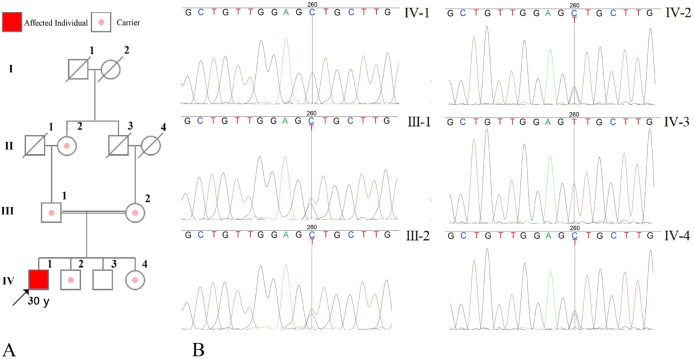
Pedigree and electropherogram of the family illustrating the inheritance of the pathogenic variant in the family; arrow shows the proband. **A** The pedigree. **B** The electeropherograms of the family members.

This study was conducted at the Comprehensive Medical Genetics Center, Shiraz, Fars Province, Iran. The proband and his parents were physically examined. Peripheral blood samples were collected for whole-exome sequencing (WES), Sanger sequencing, and other investigations. Further imaging and laboratory workups were conducted if considered necessary. Written informed consent was obtained from each subject individually or, in the case of minors, from their parents. This report is written in compliance with the CARE Statement^6^.

Genomic DNA were extracted from white blood cells using a QIAamp DNA Blood Mini Kit. WES was performed on the DNA of the patient’s white blood cells using a HiSeq 3000/4000 SBS Kit.

The raw data, converted by HiSeq X, were aligned against the human reference genome (hg19) by the Burrows–Wheeler Aligner^7^. Single-nucleotide polymorphisms were called by the Genome Analysis ToolKit software. Variants were annotated using ANNOVAR^8^. All variants were classified into five categories, namely, pathogenic, likely pathogenic, variants of unknown clinical significance, likely benign, and benign, based on the ACMG-recommended standards for the interpretation of sequence variations. The phenotypic features associated with the candidate genes were compared with the patient’s phenotype. The core phenotypes of the mutations were obtained from the OMIM database and utilized to acquire a gene list of the virtual panel from the OMIM database (OMIM # 616263).

We confirmed the presence of the pathogenic variant by Sanger sequencing. In order to amplify the mutated sites in the genome, PCR was conducted. The primers were designed using Oligo Primer Designer^9^.

The proband is a 30-year-old male. The first-degree relatives of the proband, namely, his parents, two brothers, and one sister, were examined for any problems related to the topic of this study. The proband was examined thoroughly. Additionally, peripheral blood samples were collected from each subject for further studies. Written informed consent was obtained from each patient or, in the case of minors, from their parents. The proband was the product of an uneventful pregnancy and a vaginal delivery. His mother reported difficulties in birth. His birth weight was 4800 g, and he was described as full term. The initial screenings indicated that he had G6PD deficiency. He experienced a few seizures when he was 2 days old and received medication for epilepsy. The treatment was discontinued after a year due to the spontaneous resolution of seizures. From his very early days, problems were recognized with the movement of his leg. He was able to sit with support at the age of 1 year. Following a delay, he started to walk at the age of 4 years; however, he could not walk independently until 13 years of age, and only with a walker. He was never able to walk completely unaided, and he had movement problems related to his lower limbs. Although his family claims that he was never intellectually disabled, he attended Schools for Children with Special Needs and Intellectual Disabilities for elementary and middle school. He was encouraged to attend a standard school afterward. He was able to finish secondary school. Presently, no intellectual disability is noticeable. Additionally, sensorineural hearing loss and no significant delay in language development or puberty could be observed. The patient’s motor skills, such as gripping, were impaired until the age of 12 years. However, this condition subsided afterward, and no severe complaint is currently observed. He can use his hands flawlessly, and the muscle tone in his hands is intact, with no gross deformity. The patient had a past medical history of urolithiasis. A murmur between S1 and S2 was heard in the cardiac examination. He also had severe myopia (the degree of myopia is reported to be above −6.00 diopters). He does not have any history of gastrointestinal problems, issues related to pancreatic insufficiency, or peripheral neuropathy.

The chief concern that motivated him to seek medical care was spastic diplegic cerebral palsy accompanied by tight quadriceps and pes plantus. He was able to walk only with a walker and had scissors gait and equinus foot deformity. His problems could be attributed to a novel missense pathogenic variant in the *PTRH2* gene (NM_016077.5: c.68T > C, p.V23A), as indicated in the WES report. This pathogenic variant was not reported in the ClinVar or gnomAD exomes. The homozygous presence of the pathogenic variant was detected only in the affected member of the family (the proband).

Previously, he had undergone Achilles tendon lengthening and hamstring lengthening surgery. Although his gait was improved, he still had difficulties, and his knee extensors were tight and spastic. He presented with a popliteal angle of 60 degrees in the right knee and 75 degrees in the left knee; a positive Duncan–Ely test; clonus in both legs; and muscle strength of four, four, three, and four for the right quadriceps, left quadriceps, right hamstring, and left hamstring, respectively, when admitted for surgery. Briefly, for correction, he underwent an operation involving the relocation of the right rectus femoris muscle to the posterolateral knee. A long leg cast for the right leg was subsequently applied. He also received abobotulinum toxin A (Dysport^Ⓡ^; Ipsen Pharma, Ettlingen, Germany) injected into the left quadriceps muscle. The procedure was carried out under satisfactory general anesthesia. His Gross Motor Function Classification System score improved from three to one after surgery. He is currently receiving rehabilitation therapy following surgery.

The phenotypic spectrum arising from disruptions in this protein is highly variable, to the extent that the same single-nucleotide variation can result in vastly different symptoms (Sharkia et al.^10^), (Doe et al.^11^), (Le et al.^12^). Valine 23 is a conserved amino acid (NR: 5.5799 by GERP)^13^. The variation observed in this study is categorized as damaging by DANN^14^, MutationTaster^15^, Mutation Assessor^16^, FATHMM-MKL^17^, and FATHMM-XF^18^. Although the mutation is predicted to be damaging by prediction tools, it seems that due to the similar nature of valine and alanine, the damage resulting from the disruption has only minimal effects. As a result, we propose that, in contrast to previously reported cases, the symptoms reported here are present due to disruptions in the most sensitive pathways related to PTRH2.

The symptoms reported in our study are different from those reported in previous studies. Although movement disorders, motor delay, and severe myopia were present, the gastrointestinal examination was unremarkable, and facial dysmorphism and hearing disorders were not present^10–12^. In light of the great differences between the presentation of PTRH2 deficiency in the current case and in previously reported cases, further research to elucidate the cause of these differences is highly advisable.

We propose that further studies are needed to confirm the pathogenicity of this variant. Although the proband’s movement-related symptoms are similar to those in previously reported cases, a number of other symptoms are missing, which can be attributed to the presumed mild effect of this pathogenic variant on the function or transport of PTRH2. With regard to the importance of the pathogenic variants of this gene and the phenotypic spectrum of patients, the variant should be actively searched for, further studied, and clinically characterized. The main challenge that arises regarding the results of our study is the detection of this variant in prenatal diagnosis, which necessitates new studies to cast light on the phenotypic spectrum and new variants that could cause a new set of symptoms ranging from mild to severe. With such information, decisions regarding abortion or continuation of pregnancy could be made on a more robust basis in the future.

## Data Availability

The relevant data from this Data Report are hosted at the Human Genome Variation Database at 10.6084/m9.figshare.hgv.2987.

## Data availability

All data generated or analyzed during this study are included in the final published article.

## References

[1] Menninger JR (1976). Peptidyl transfer RNA dissociates during protein synthesis from ribosomes of Escherichia coli. J. Biol. Chem..

[2] Jan Y (2004). A mitochondrial protein, Bit1, mediates apoptosis regulated by integrins and groucho/TLE corepressors. Cell.

[3] de Pereda JM, Waas WF, Jan Y, Ruoslahti E, Schimmel P, Pascual J (2004). Crystal structure of a human peptidyl-tRNA hydrolase reveals a new fold and suggests basis for a bifunctional activity. J. Biol. Chem..

[4] Hu H (2014). Mutations in PTRH2 cause novel infantile-onset multisystem disease with intellectual disability, microcephaly, progressive ataxia, and muscle weakness. Ann. Clin. Transl. Neurol..

[5] Alazami AM (2015). Accelerating novel candidate gene discovery in neurogenetic disorders via whole-exome sequencing of prescreened multiplex consanguineous families. Cell Rep..

[6] Gagnier JJ (2013). The CARE guidelines: consensus-based clinical case reporting guideline development. Glob. Adv. Heal. Med..

[7] Li H, Durbin R (2010). Fast and accurate long-read alignment with Burrows-Wheeler transform. Bioinformatics.

[8] Wang K, Li M, Hakonarson H (2010). ANNOVAR: functional annotation of genetic variants from high-throughput sequencing data. Nucleic Acids Res..

[9] Rychlik W (2007). OLIGO 7 primer analysis software. Methods Mol. Biol..

[10] Sharkia R (2017). Homozygous mutation in PTRH2 gene causes progressive sensorineural deafness and peripheral neuropathy. Am. J. Med. Genet Part A..

[11] Doe J (2017). PTRH2 gene mutation causes progressive congenital skeletal muscle pathology. Hum. Mol. Genet.

[12] Le C (2019). Infantile-onset multisystem neurologic, endocrine, and pancreatic disease: case and review. Can. J. Neurol. Sci./J. Can. des. Sci. Neurol..

[13] Davydov EV, Goode DL, Sirota M, Cooper GM, Sidow A, Batzoglou S (2010). Identifying a high fraction of the human genome to be under selective constraint using GERP++. PLoS Comput. Biol..

[14] Quang D, Chen Y, Xie X (2015). DANN: a deep learning approach for annotating the pathogenicity of genetic variants. Bioinformatics.

[15] Schwarz JM, Cooper DN, Schuelke M, Seelow D (2014). MutationTaster2: mutation prediction for the deep-sequencing age. Nat. Methods.

[16] Frousios K, Iliopoulos CS, Schlitt T, Simpson MA (2013). Predicting the functional consequences of non-synonymous DNA sequence variants—evaluation of bioinformatics tools and development of a consensus strategy. Genomics.

[17] Shihab HA (2015). An integrative approach to predicting the functional effects of non-coding and coding sequence variation. Bioinformatics.

[18] Rogers MF (2017). FATHMM-XF: accurate prediction of pathogenic point mutations via extended features. Bioinformatics.

